# Circulating Tumor DNA as a Prognostic and Predictive Biomarker in Lung Cancer

**DOI:** 10.3390/cancers17203327

**Published:** 2025-10-15

**Authors:** Puneet Dhillon, Simo Du, Haiying Cheng

**Affiliations:** 1 Department of Oncology, Albert Einstein College of Medicine/Montefiore Medical Center/Montefiore Einstein Comprehensive Cancer Center, Bronx, New York, NY 10461, USA; pudhillon@montefiore.org; 2 Department of Internal Medicine, NYC Health + Hospitals/Jacobi Medical Center, Bronx, New York, NY 10461, USA; dus1@nychhc.org

**Keywords:** ctDNA, lung cancer, NSCLC, cfDNA, NGS, precision medicine, liquid biopsy

## Abstract

**Simple Summary:**

Liquid biopsy is a type of blood test which detects circulating tumor DNA (ctDNA). This is a key advance in precision medicine which can help in diagnosing, treating, and monitoring lung cancer. This article reviews the current applications of ctDNA in the context of lung cancer, particularly non-small cell lung cancer (NSCLC). Research to date reveal ctDNA is useful in detecting actionable genomic alterations, helps guide treatment, and may predict how the cancer may respond to treatment and evolve. Minimal residual disease detection is another key area of interest. ctDNA detection and tracking have powerful potential uses, and many studies are currently underway.

**Abstract:**

**Background/Objectives:** Lung cancer remains a leading cause of cancer-related mortality worldwide. In recent years, the development of liquid biopsy, or ctDNA detection in body fluids, particularly blood, has been shown to be effective in detection, genotyping, prognostication, and evaluating therapy response, particularly in non-small cell lung cancer (NSCLC). **Methods:** In this review, we present a summary of the current landscape of ctDNA, applications, and limitations, as well as future areas of research. **Results/Conclusions:** Though not yet in its prime, ctDNA detection and tracking have powerful current and potential uses, including treatment selection, prognostication, and risk stratification.

## 1. Introduction

Lung cancer is one of the most diagnosed cancers worldwide at 12.4% of total cancer diagnoses [1] and the second most diagnosed cancer in the United States in both men and women. It remains one of the leading causes of cancer-related deaths, causing approximately 131,584 deaths in the United States alone in 2023 [2].

Around 85% of lung cancer is non-small cell lung cancer (NSCLC) [3]. In recent years, the discovery of driver mutations in NSCLC and the utilization of mutation-targeted therapies have significantly improved therapeutic options for patients. Due to this, tumor genotyping is of the utmost importance and is traditionally performed on specimens that are obtained through invasive means, like tissue biopsies. Liquid biopsy is a non-invasive method which analyzes circulating tumor DNA (ctDNA) and can assist in genotyping the tumor [4,5,6].

Cell-free DNA (cfDNA) comprises partially degraded DNA fragments that are not encased in cells. Circulating tumor DNA (ctDNA) in particular comprises short, double-stranded DNA fragments that are released by tumor cells into blood and, depending on the tumor, other biologic fluids like cerebrospinal fluid or urine. ctDNA shed from cancer cells holds unique genetic alterations from where the tumor originated [7,8].

Various international oncology societies currently recommend the use of liquid biopsy for tumor molecular genotyping in lung cancer.

Liquid biopsy is a term used to describe sampling of body fluid, typically blood, to detect tumor cells and cancer cell DNA [6,8]. There are multiple advantages, including being minimally invasive. ctDNA is also thought to better reflect intra-tumoral heterogeneity, given the ability to capture information from different tumor sub-clones. The turnaround time of ctDNA detection is relatively quick, and it can be performed in the office without needing to plan for a biopsy [9,10].

There are various methods to detect ctDNA. Currently, ctDNA testing is typically performed using polymerase chain reaction (PCR)-based or next-generation sequencing (NGS), though NGS is more commercially utilized [11,12,13]. Digital PCR-based methods detect specific DNA sequences and have been preferred in this context over prior PCR methods, but they interrogate only specific numbers of mutations at a time and cannot easily detect copy number alterations and rearrangements [14,15,16]. TAM-seq and CAPP-seq are NGS-based assays. They retain high sensitivity and specificity and can detect multiple mutations of interest, as well as copy number alterations [17,18]. However, for NGS assays, turnaround times can be longer, the cost is higher than for PCR, and they require bioinformatics result interpretation [16,17,18,19].

Fragmentomics analyzes the size patterns of cfDNA and is another area of ongoing research [20,21,22]. Methylation patterns of ctDNA can mirror those in cancerous tissue, and studies have been investigating applications in detection and monitoring [23].

Most commonly, ctDNA testing is performed on blood samples, but it can also be performed in cerebrospinal fluid (CSF) or body fluid; for example, pleural fluid ctDNA analysis of patients with brain metastasis has revealed either the absence or very low levels of ctDNA, possibly because of the blood–brain barrier [24]. However, one study found that ctDNA in CSF had a higher sensitivity than plasma for CNS genomic alterations [25], and another study found that the EGFR mutation detected was the same in the CSF and blood [26,27]. Other body fluids have also been studied, such as urine, saliva, and bronchoalveolar lavage fluid, but they do not have clinically relevant applications yet [28,29,30,31].

In NSCLC, ctDNA has been evidenced to be effective in detection, genotyping, and evaluating therapy response, though further work is required in establishing standardized practices.

## 2. Applications

### 2.1. Detection/Screening

In patients who qualify for lung cancer screening, low-dose CT is recommended for early detection, but there are suboptimal compliance rates due to cost and difficulty [20]. Detection of cancers with ctDNA is being studied as a tool to aid in screening to augment low-dose CT chest screening [32,33]. Limitations include early-stage cancers being harder to detect as ctDNA level has been shown to be proportional to tumor burden [31,32,34,35]. There has been work on testing machine learning models to diagnose lung cancer, combining ctDNA, imaging, patient risk factors, and fragmentation features [20,36].

ctDNA levels can fluctuate in response to treatment and thus can aid in monitoring disease progression and/or recurrence. Overall, there is a positive correlation with tumor burden, and higher ctDNA levels in the blood have been linked to poorer prognosis in NSCLC [37,38,39]. There are various clinical trials that aim to study the role of ctDNA in lung cancer screening and stratification such as the Lung Cancer Mutation Consortium (NCT04712877) and DNA Evaluation of Fragments for Early Interception—Lung Cancer Training Study (DELFI-L101 Study) (NCT04825834) [24].

### 2.2. Genotyping and Detecting Actionable Genomic Alterations (AGAs)

**Figure 1:**
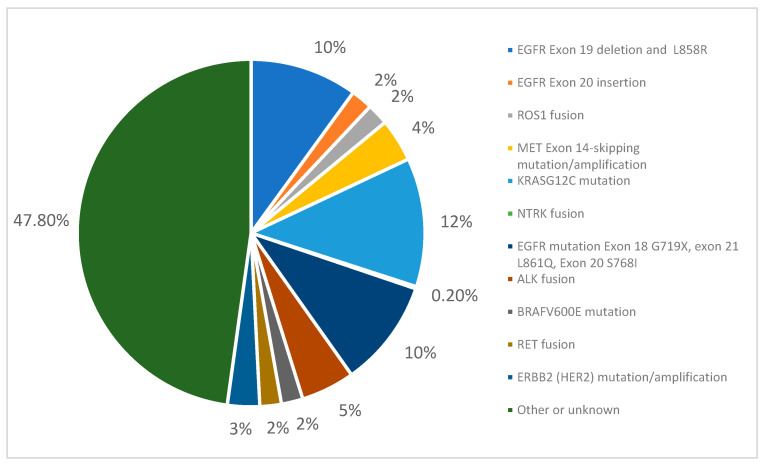
The prevalence of various actionable genomic alterations in adenocarcinoma. Of note, the prevalence of EGFR exon 19 deletions and EGFR Exon 20 mutation L858R occurs at a higher rate in ~50% of the Asian population, and may vary amongst different populations.

ctDNA can be used to identify specific mutations in lung cancer and guide selection of targeted therapy, which has significantly improved outcomes, especially in recent years [32]. It is current practice to test for EGFR, KRAS, ALK, ROS1, BRAF, NTRK 1/2/3, MET, RET, ERBB2 (HER2), and NRG1 via liquid or tissue biopsy at diagnosis, recurrence, and/or progression. Figure 1 shows the prevalence of different mutations in lung adenocarcinoma, which makes up about 45% of NSCLC. Around 65% of these patients may harbor a clinically actionable genomic alteration [1,2,3,4,5,6,40].

Molecular genotyping from liquid biopsy can be as effective as from a tissue specimen, depending on the specimen characteristics. Specific genomic alterations can also be monitored longitudinally, like in copy number mutations over time, like EGFR after surgery. Liquid biopsy can be performed at any stage of disease including at the time of progression to evaluate for any new potential actional genomic alterations to guide treatment decisions [15,41,42].

There has been some evidence of increased adherence to standard of care and overall more prompt care, though this relationship is likely not causal. For instance, Thompson et al. demonstrated that the inclusion of plasma-based NGS testing led to higher rates of guideline-recommended treatment (74% vs. 46%, *p* = 0.0005), but it was noted that physicians had test results available before the first patient visit much more frequently when a plasma-based strategy was used, which is likely a contributing factor [43].

### 2.3. Utilizing ctDNA in Resectable Disease

Approximately 25% to 30% of patients with NSCLC present at stages I to IIIA [11,12,13]. Surgery remains the definitive treatment for early-stage NSCLC. Unfortunately, 30–50% of NSCLC patients will relapse after surgery. Adjuvant treatment has improved overall survival (OS) by around 5%, but a significant proportion of patients develop recurrence, resulting in a median OS rate ranging from 84% at stage IA to 36% at stage IIIA. Minimal residual disease (MRD) refers to the small number of remaining cancer cells, or micrometastasis, during the course or after the completion of treatment. An effective strategy to eradicate MRD and therefore minimize the chance of post-operative recurrence consists of adjuvant therapy. Currently disease is followed using imaging and monitored using the Response Evaluation Criteria in Solid Tumors (RECIST) criteria. CT imaging is most commonly used, and small lesions may be missed given the limit of detection is 2–3 mm. Using ctDNA to track MRD and perform appropriate risk stratification and treatment decisions is an area of research which is becoming more studied.

ctDNA MRD analyses can be tumor-informed or tumor-agnostic [44] and depend on prior knowledge of tissue sequencing. Tumor-informed analysis requires sequencing of tumor tissue to identify mutations to track and is more sensitive. One of the main approaches used has been to employ NGS to track mutations since this facilitates the tracking of multiple mutations in parallel, minimizes the number of tests needed, and increases sensitivity [34], but it requires longer assay development times and may not capture newly evolved mutations [33,45]. Tumor-agnostic methods utilize epigenetic features such as DNA methylation and DNA fragmentation patterns to detect ctDNA. Currently, the sensitivity of these methods is worse compared to tumor-informed approaches and thus, at this time, they are less applicable in MRD tracking [45].

Studies using a variety of tumor-informed NGS-based ctDNA MRD assays have demonstrated that the detection of residual ctDNA after completion of treatment means a higher risk of recurrence [11,12,13,39,45]. There has been variation among them in terms of when ctDNA is drawn after definitive treatment. Lower ctDNA shedding has also been associated with the adenocarcinoma histological subtype as opposed to squamous cell carcinoma [13].

There are two main methodologies for the MRD testing timeline—longitudinal or serial measurements over time and landmark or singular designated time points [19]. Though there is no clear recommendation for one methodology, studies will typically utilize one of the other. In a systematic review of 13 studies that performed ctDNA analysis for post-operative MRD detection in stage I-III NSCLC patients, higher overall sensitivity was achieved in studies that performed longitudinal MRD analysis compared to landmark analysis [18,19].

Chauduri et al. showed that longitudinal tracking of multiple mutations can significantly increase the sensitivity of post-treatment MRD detection (94% vs. 58% for a single mutation *p* = 0.001) [11,12]. In a prospective study by Zhang et al., landmark analysis was utilized at one month post-operatively and at one month post-adjuvant therapy. For those who received adjuvant chemotherapy, 86.6% of patients with negative MRD status remained disease-free [39]. Different studies utilize different time points in both methodologies, but obtaining measurements at baseline, post-operatively, and before and after systemic therapy seems pertinent, as these landmark timepoints are clinically relevant, though more trials are needed [39].

One of the key applications of interest in MRD testing is guiding treatment decisions on when to de-escalate treatment, such as in decisions around adjuvant therapy.

#### 2.3.1. Chemoimmunotherapy in Resectable Disease

Neoadjuvant chemotherapy and/or immunotherapy is an important treatment strategy in resectable NSCLC [46]. The phase II NADIM study that included patients with resectable stage IIIA disease showed that clearance of ctDNA after three cycles of neoadjuvant nivolumab plus chemotherapy was significantly correlated with longer progression-free survival (PFS) (HR: 0.16, 95% CI: 0.03–0.73) and overall survival (OS) (HR: 0.05, 95% CI: 0–0.62). It is of note that analyses excluded patients with undetectable ctDNA at baseline [47].

The phase III AEGEAN trial used peri-operative durvalumab plus neoadjuvant chemotherapy in patients with resectable stage II-IIIB NSCLC and showed that patients achieving ctDNA clearance after three cycles of neoadjuvant therapy had significantly better event-free survival (EFS) outcomes compared with those with residual ctDNA, especially in the durvalumab-plus-chemotherapy arm (HR: 0.26; 95% CI: 0.13–0.54) [48]. Patients with no detectable ctDNA before surgery had better survival outcomes even in the absence of pathologic complete response [48,49].

It is important to note that the negative prognostic value of ctDNA clearance is suboptimal at this time. Analyses from CheckMate 816 (neoadjuvant nivolumab plus chemotherapy) and CheckMate 77T (peri-operative nivolumab plus chemotherapy trials in resectable NSCLC) both showed that although clearance of ctDNA was correlated with a higher pCR rate, as many as 50% of patients with ctDNA clearance did not achieve pCR, implying that ctDNA negativity may not necessarily translate into eradication of residual tumor cells [50].

#### 2.3.2. Targeted Therapies in Resectable Disease

Targeted therapies have significantly changed the landscape of treatment for EGFR-mutated NSCLC in general, though it is not yet clear how MRD can guide therapy duration and personalize treatment to patients. Herbst et al. in post hoc ADAURA trial analysis looked at patients that would benefit beyond three years using MRD to guide therapy. In this study, MRD preceded DFS events in most patients [51]. DFS and MRD event-free status was maintained for most patients during adjuvant osimertinib treatment and posttreatment follow-up, with most MRD or DFS events occurring after osimertinib treatment discontinuation or completion [52].

The TRACERx study used ctDNA to track a median of 200 mutations in resected NSCLC tissue. It utilized a tumor-informed approach (anchored multiplex PCR) to detect MRD in early-stage NSCLC after surgery. It found that 25% of patients were positive for MRD within 120 days of surgery, and 3- to 6-monthly ctDNA surveillance identified relapse in a further ~20% of landmark-ctDNA-negative patients. A bioinformatic tool, ECLIPSE, was used to track sub-clonal architecture at low ctDNA levels, which identified patients with polyclonal metastatic dissemination and was associated with poor clinical outcomes [51,53].

Jung et al. studied stage I–IIIA EGFR-mutated NSCLC patients who underwent curative resection. ctDNA was measured prior to surgery, after curative surgery, and then at different time points with increasing interval lengths up to 5 years or until clinically definite recurrence [54]. Patients were then classified into three groups: baseline ctDNA-negative, baseline ctDNA-positive but post-operative MRD-negative, and baseline ctDNA-positive and post-operative MRD-positive. The study found that the 3-year DFS rate was significantly different among the three groups (84% versus 78% versus 50%, *p* = 0.02), suggesting the possible future development of ctDNA MRD status-based risk stratification [54]. Table 1 shows a concise summary of key studies highlighting resectable NSCLC and ctDNA applications and outcomes.

### 2.4. Locally Advanced, Metastatic, and Recurrent NSCLC

Like in resectable disease, tracking changes using RECIST criteria represents the primary quantification of response and progression [55,59,62,63,64]. In advanced disease, the utility of ctDNA lies in the ability to track it over time to measure progression prior to radiographic progression, and, in theory, change treatment if needed, to improve patient outcomes. Additionally, ctDNA tracking after chemoradiation or consolidation therapy to guide therapy is another area of interest [56,65,66,67,68]. This is not yet a guideline-directed application.

Studies have shown that detecting ctDNA after chemoradiation is associated with worse prognosis, prompting further investigation [69]. Moding et al. studied samples from patients treated with chemoradiation for locally advanced NSCLC, including patients receiving consolidation immunotherapy. Patients ctDNA-negative after chemoradiation had better outcomes, and outcomes of those with MRD after chemoradiation were significantly improved by consolidation immunotherapy [69]. In a prospective observational study, Gale et al. found that patients with ctDNA negativity in all plasma samples and those with ctDNA clearance (previously positive) had longer PFS and OS than patients with persistent ctDNA detection [57]. In certain therapeutic scenarios, the relationship of ctDNA may help guide one towards a specific therapeutic [70,71,72]. For patients being treated for advanced disease with amivantamab plus lazertinib, there have been improved outcomes in high-risk patients with baseline positive ctDNA, as well as without ctDNA clearance, compared to osimertinib [73].

Some studies demonstrate the importance of a molecular response in the relevant biomarker or molecular target irrespective of the target. In metastatic HER2 NSCLC, plasma ctDNA was tested before first-line therapy, and it was found that patients with detectable baseline ctDNA had significantly shorter OS (hazard ratio (HR), 5.25; 95% CI, 1.2–23.9; *p* = 0.019) [74]. The phase II VISION study evaluated tepotinib in patients with advanced or metastatic NSCLC with a confirmed MET exon 14 skipping mutation and found a high concordance between the molecular cfDNA response and clinical response based on RECIST tumor measurement. Four patients who had decreased levels of cfDNA during treatment did have clinical progression [75]. In patients with KRASG12C-mutated NSCLC treated with sotorasib, those with detectable KRASG12C had inferior progression-free survival (hazard ratio [HR] 2.13 [95% confidence interval [CI]: 1.06–4.30], p ¼ 0.031) and overall survival (HR 2.61 [95% CI: 1.16–5.91], p ¼ 0.017). The disease control rate was significantly higher in those with a molecular response. KRAS amplifications were identified as recurrent treatment-emergent alterations which represent a proposed mechanism of resistance [76].

Table 2 shows a concise summary of key studies highlighting advanced NSCLC and ctDNA and a summary of relevant outcomes. In short, ctDNA detection after an intervention may be a prognostic indicator.

### 2.5. Resistance

Detecting resistance early to guide treatments and evaluate if there is an impact on outcomes is also a purpose for which ctDNA is being studied [84,87,88,89]. Liquid biopsy is particularly effective in detecting genotypic variations in the background of clonal drift and molecular heterogeneity.

Most patients treated with first- and second-generation TKIs develop resistance early [16,17]; osimertinib is a preferred TKI for this reason [79,81,90,91,92]. However, resistance to osimertinib does occur, and knowing resistance patterns may help predict the course of treatment. The most common mechanism of resistance to osimertinib (which inhibits the T790M isoform) is the development of the C797S isoform, as well as L792X, G796S, L718Q, S768I, G796R, G796D, and G724S [40,93,94]. EGFR gene amplifications and copy number alterations are also mechanisms of resistance [95]. Other mechanisms of resistance include novel fusion events, ERBB2 amplification, and activation of the RAS-MAPK or RAS–PI3K pathway [96,97]. Kato et al. in 2021 performed deep sequencing (CAPP-seq) to analyze ctDNA and were able to identify potentially targetable genetic alterations in patients with osimertinib resistance [43].

Yamaguchi et al. evaluated ctDNA during osimertinib administration as a second-line or further treatment to identify the relationship between EGFR mutation levels and outcomes in patients with advanced NSCLC. In patients with EGFR T790M-positive NSCLC who were receiving osimertinib after prior EGFR-TKI treatment, ctDNA was collected at pretreatment, after 1 month, and at the time of POD. The detection rate of copy numbers of exon 19 deletion, L858R, and T790M in plasma samples was significantly lower 1 month after osimertinib than at pretreatment and significantly higher at PD than at 1 month, whereas that of C797S was significantly higher at PD than at 1 month. No statistically significant difference was observed in the copy numbers of exon 19 deletion, L858R, T790M, and C797S between complete response or partial response and stable disease or PD. Testing based on ctDNA may be helpful in predicting outcomes of osimertinib treatment in T790M-positive NSCLC after previous EGFR-TKI treatment [84].

## 3. Future Directions

Though there are promising data, further studies are paramount in evaluating the role of using ctDNA to guide care and therapeutic decisions in NSCLC in a way that effects patient outcomes. Both resectable and advanced NSCLC are areas that have seen several promising studies showing prognostic and predictive value, and there are myriad clinical trials that will hopefully help guide clinicians on when and how to change therapeutic course and impacts on survival. Particularly, the ability to risk-stratify patients and escalate or de-escalate based on ctDNA detection would be paramount in limiting toxicities and improving clinical outcomes. Using ctDNA in screening is still immature but could augment low-dose CT and, in the future, could aid in risk stratification. We hope that further studies in these directions lead to consistent incorporation and recommendations for clinicians.

## 4. Conclusions

The development of liquid biopsy has been a significant development, especially to detect actionable genomic alterations and to choose the appropriate therapy. Though ctDNA has clear indications in guidelines for detecting key biomarkers and has promising applications, it is not uniformly integrated into care and has not reached its full potential yet. The sensitivity and negative predictive value of current ctDNA testing are still low, especially for early-stage cancers. Further research is needed to standardize ctDNA analysis and validate its clinical utility for lung cancer screening, diagnosis, and prognostication.

Additionally, variants can be detected that are not related to the tumor [clonal hematopoiesis of indeterminate potential (CHIP)]. It is also important to note that there are no standard approved practices for MRD testing at this time. There are numerous ongoing clinical trials to evaluate the value of ctDNA in various stages and clinical applications, and ctDNA is effective in the clinical setting so far. Table 3 shows a list, including primary outcomes.

## Figures and Tables

**Table 1:** Key studies following or utilizing ctDNA in resectable NSCLC.

Study	Study Design, Stage, and/or Population	Outcome
Guo et al., 2016 [55]	Prospective; all stages	ctDNA from stage IA and IB patients had more dramatic decrease post-op compared with mutation frequency in more advanced cancer pts
Abbosh et al., 2017 [13]	Prospective; IIA–IIIB	Tumor volume correlated with the mean plasma VAF of clonal SNVs in ctDNA-positive NSCLCs
Chaudhuri et al., 2017 [11]	Retrospective; IB–IIIB,	Posttreatment ctDNA detection preceded radiographic progression in 72% of patients by a median of 5.2 months
Chen et al., 2019 [39]	Prospective; I–IIIA	Rapid decrease in ctDNA occurred after radical tumor resection. Median ctDNA half-life was 35 min. RFS in pts with detectable ctDNA was shorter than in those without
Waldeck et al., 2022 [56]	Prospective; IA–IIIB	Of ctDNA-negative pts, 33% experienced relapse post-op. Positive ctDNA in early post-op plasma samples was associated with shorter PFS and OS
Gale et al., 2022 [57]	Prospective; IA–IIIB	ctDNA detection had clinical specificity >98.5% and preceded clinical recurrence by a median of 212.5 days
Abbosh et al., 2023 [53]	Prospective; II–III	Results showed that 3- to 6-monthly ctDNA surveillance identified impending disease relapse in 20% of landmark-negative patients
Jung et al., 2023 [54]	Prospective; I-IIIA EGFR mutant	Of patients with baseline +ctDNA, 76% had clearance at 4 weeks after surgery. MRD was detected before radiological recurrence in 69% of patients with exon 19 deletion and in 20% with L858R mutation
Provencio et al., 2022 [47]	Phase II trial; IIIA or IIIB	ctDNA levels were associated with differences in PFS/OS; 67% of the experimental group and 44% of the control group were ctDNA-negative after neoadjuvant treatment.
Oh et al., 2024 [58]	Retrospective; I–IV	Pts with ctDNA+ were more likely to experience recurrence compared to ctDNA- patients. ctDNA+ was associated with poorer RFS than persistently ctDNA- patients
Ohara et al., 2020 [59]	Prospective; II–III	ctDNA+ pts had disease recurrence within median of 9.1 months and shorter RFS/OS than those without detectable ctDNA. Three pts who had CNS-only metastases did not have detectable ctDNA
Tan et al., 2024 [60]	Retrospective; I–III	ctDNA detection preop was associated with shorter RFS. ctDNA positivity preceded radiological findings by a median lead time of 2.8 months
Herbst et al., 2022 [51]	Phase III post hoc; IB–IIIA; EGFR mutant	MRD+ had clinical sensitivity of 65% and specificity of 95% and preceded a DFS event by a median of 4.7 months
Rosenlund et al., 2025 [61]	Prospective; I–III	Detectable ctDNA post-treatment was significantly associated with increased risk of tumor recurrence and shorter RFS. Post RT/CRT, ctDNA detection was significantly linked to shorter RFS in MRD analysis
Schuurbiers et al., 2025 [62]	Retrospective; I–III	Before treatment, ctDNA was detected in 48% LEMA and 51% LUCID pts. ctDNA detection after treatment was associated with shorter recurrence-free survival and overall survival

**Table 2:** Outlines of key studies on ctDNA and outcomes in advanced and metastatic NSCLC.

Study	Study Design, Stage, and/or Population	Outcome
Liu et al., 2024 [74]	Retrospective; Metastatic HER2-mutant	*HER2* mutations tracked longitudinally correlated with treatment response. Pts with detectable BL ctDNA had shorter OS
Song et al., 2020 [77]	Prospective; IIIB to IV	Higher ctDNA at BL was associated with shorter OS. ctDNA- was associated with longer PFS/OS regardless of type of treatment
Paik et al., 2020 [78]	Phase II; advanced/metastatic MET exon 14 skipping mutation	A total of 67% of patients had a molecular cfDNA response; of those, 71% had a radiographic response. ctDNA and imaging PD were highly concordant
Anagnostou et al., 2023 [75]	Phase II; advanced/metastatic PD-L1 expression level of ≥1%	Median time to ctDNA response was 2.1 months; pts with MR had longer PFS and OS
Jun et al., 2024 [79]	Phase II post hoc; III	ctDNA+ predicted inferior PFS after completion of CRT and at the end of CPI
Moding et al., 2020 [69]	Stage IIB-IIIB NSCLC	Pts who were ctDNA- after CRT had better outcomes regardless of CICI. Patients with MRD+ after CRT who received CICI had significantly better outcomes than pts who did not receive CICI
Pan et al., 2023 [80]	Stage IIB- IIIC	Longitudinal undetectable MRD was found in 20.1% of patients. The 2-year PFS of these pts was 88.4%
Yang et al., 2022 [81]	Stage III NSCLC	ctDNA collected 1 month post-CRT/RT was the optimal choice to predict pts’ PFS and OS, and the dynamic change in ctDNA was closely associated with clinical outcomes
Horndalsveen et al., 2025 [82] (abstract)	Phase II; III	Preliminary OS data shows that detectable ctDNA during the first four months post-CRT significantly increased the odds of death within 24 months
Ernst et al., 2024 [83]	Prospective; advanced KRASG12C-Mutated	Pretreatment, KRASG12C ctDNA was detected in 76% of pts. The disease control rate was significantly higher in those with a molecular response
Yamaguchi et al., 2023 [84]	Prospective; III-IV	Detection of T790M at PD after osimertinib initiation was a significant independent prognostic factor for predicting shorter prognosis
Shaw et al., 2019 [85]	Phase II; ALK mutation	PFS was similar in pts with and without ALK mutations on plasma genotyping but longer in patients with ALK mutations on tissue genotyping
Vendrell et al., 2021 [86]	Prospective; IV	Pts who developed an EGFR-dependent mechanism of resistance responded longer to osimertinib than EGFR-independent resistant pts
Gray et al., 2024 [9]	Phase III post hoc; advanced stage	ctDNA PD preceded or co-occurred with RECIST-defined PD: 64% in FLAURA and 56% in AURA3
Remon et al., 2023 [87]	Prospective; EGFR mutant	Molecular PD before RECIST PD led to an earlier switch to osimertinib in 17% of patients with satisfactory PFS/OS outcomes

**Table 3:** Active clinical trials using ctDNA in lung cancer, divided into resectable, non-resectable, and screening/stratification categories. All are active unless otherwise indicated by an asterisk.

Trial/Study (with NCT if Available)	Study Type	Objective/Goal
Resectable		
NCT06358222	Observational	Develop tumor-naïve MRD panel to predict nodal disease
NCT06426511	Phase II	Personalize consolidation toripalimab based on MRD
NCT06979661	Early Phase I	Use Haystack^TM^ MRD to guide post-op RT/systemic therapy
NCT05536505	Phase II	De-escalate in MRD−; treat MRD+ with icotinib/osimertinib
NCT04317534	Phase II	Observation vs. pembrolizumab in stage I with ctDNA correlatives
NCT04712877	Observational	Validate biomarker-integrated peri-operative care pathways including ctDNA
*NCT05079022	Phase I/II	Test benefits of adjuvant furmonertinib specifically in MRD+ stage I
*NCT04385368	Phase III	Determine if adding durvalumab to adjuvant chemotherapy improves DFS in MRD+ post-resection
*NCT04642469	Phase III	Test durvalumab vs. placebo in patients who become MRD+ during surveillance after curative therapy
NCT06053099	Prospective cohort	Define prognostic and mechanistic correlates of relapse during/after adjuvant osimertinib
*NCT04037150	Observational	Evaluate peri-operative ctDNA as a surveillance tool
NCT06323148	Phase III	Test adjuvant osimertinib specifically in MRD+
NCT05457049	Observational	Evaluate therapy-free surveillance if MRD remains undetectable
NCT05059444	Observational	Validate a novel commercial ctDNA assay for surveillance
*NCT04351555	Phase III	Evaluate benefits of MRD in neoadjuvant osimertinib ± chemo vs. chemo
NCT04966663	Phase II	Test adjuvant therapy in ctDNA+ vs. observation
NCT05167604	Observational	Explore MRD status and correlation with outcomes after adjuvant chemo in early NSCLC
NCT04367311	Phase II	Assess intensified adjuvant chemo + atezolizumab for post-op ctDNA+ pts
*NCT04625699	Phase II	Evaluate peri-operative IO doublet for patients who develop detectable ctDNA after standard adjuvant therapy
NCT05236114	Observational	Map optimal peri-op timepoints for ctDNA MRD
NCT04638582	Phase II	Define benefits of preop pembrolizumab with ctDNA correlatives in early NSCLC
NCT05254782	Observational	Quantify detection rate and prognostic value of peri-operative ctDNA
NCT05460195	Phase II	Peri-op sintilimab and anlotinib based on MRD
NCT04585477	Phase II	ctDNA-adapted adjuvant immunotherapy strategies
Advanced/Metastatic		
NCT04841811	Phase III	Evaluate almonertinib in unresectable stage III EGFRm NSCLC; evaluate dynamic MRD-guided maintenance therapy with almonertinib
*NCT06020989	Phase II	Determine if early chemo add-on for ctDNA-positive patients improves outcomes vs. lazertinib alone
NCT04912687	Observational	Implement and evaluate baseline ctDNA testing at initial diagnosis
NCT05334277	Phase II	Test ctDNA-guided escalation after furmonertinib induction
*NCT03865511	Phase II	Characterize on-treatment resistance using paired biopsies and ctDNA with osimertinib
NCT04737382	Observational	Define concordance and clinical utility of tumor NGS vs. plasma ctDNA
NCT05281406	Phase II	Assess benefit of adding platinum + pemetrexed for early ctDNA persistence on 1 L osimertinib
NCT05598528	Observational	Identify predictors of primary resistance to 3rd-gen EGFR TKIs
NCT05257967	Observational	Quantify incremental value of CSF ctDNA in leptomeningeal disease
NCT05401110	Phase I	Determine safety/activity of carotuximab + osimertinib; follow ctDNA+
NCT05534113	Phase II	Evaluate sequential envafolimab after ctDNA clearance on almonertinib
NCT05813522	Phase II	Assess high-dose furmonertinib efficacy in LM and validate CSF ctDNA monitoring
NCT04585490	Phase III	Personalize consolidation durvalumab after CRT using ctDNA
NCT05198154	Observational	Anticipate progression using ctDNA in long-responders
Screening/Risk Stratification		
*NCT05117840	Observational	Evaluate blood-based GuardantLUNAR-2 screening assay
NCT06163846	Observational	Integrate imaging and liquid biopsy for stratification
*NCT05020275	Observational	Map resistance to inform subsequent therapy
NCT03774758	Observational	Validate ctDNA signature for risk stratification
*NCT02194738	Screening (ALCHEMIST)	Test resected NSCLC for genetic mutations to facilitate accrual to randomized adjuvant studies of adjuvant treatment trials for resected NSCLC

## Data Availability

No datasets were generated or analyzed during the current study.

## Abbreviations

The following abbreviations are used in this manuscript:

AGA	Actionable genomic alterations.
BL	Baseline.
CFDNA	Cell-free DNA.
CICI	Consolidation immune checkpoint inhibition.
CNS	Central nervous system.
CPI	Checkpoint inhibitor.
CRT	Chemoradiation therapy.
CTDNA	Circulating tumor DNA.
DFS	Disease-free survival.
DNA	Deoxyribonucleic acid.
EGFR	Epidermal growth factor receptor.
ICI	Immune checkpoint inhibition.
MPR	Major pathologic response.
MR	Molecular response.
MRD	Minimal residual disease.
NSCLC	Non-small cell lung cancer.
OS	Overall survival.
ORR	Overall response rate.
pCR	Pathologic complete response.
PD	Progression of disease.
PFS	Progression-free survival.
PT/PTs	Patient/Patients.
RECIST	Response Evaluation Criteria in Solid Tumors.
RFS	Recurrence-free survival.
RT	Radiotherapy.
TKI	Tyrosine kinase inhibitors.
USPSTF	United States Preventive Services Taskforce.
